# Treating Spontaneous Pneumothorax Using an Innovative Surgical Technique Called Capnodissection Pleurectomy: Case Report

**DOI:** 10.2196/54497

**Published:** 2024-06-21

**Authors:** Ghaith Qsous, Prashanth Ramaraj, Sanjeet Singh Avtaar Singh, Philip Herd, Nayandra Runveer Sooraj, Malcolm Brodie Will

**Affiliations:** 1 Department of Cardiothoracic Surgery Royal Infirmary of Edinburgh Edinburgh United Kingdom; 2 Imperial College School of Medicine Imperial College London London United Kingdom; 3 Department of Anaesthesia Royal Infirmary of Edinburgh Edinburgh United Kingdom

**Keywords:** capnodissection, pleurectomy, VATS, video-assisted thorascopic surgery, novel technique, thoracic surgery, surgical innovation, pneumothorax, spontaneous pneumothorax, pleurodesis, management, bullectomy, bullae, young patient, lung diseases, chronic obstructive pulmonary disease, COPD, surgical treatment, male, capnothorax

## Abstract

Spontaneous pneumothorax is one of the most common conditions encountered in thoracic surgery. This condition can be treated conservatively or surgically based on indications and guidelines. Traditional surgical management includes pleurodesis (mechanical or chemical) in addition to bullectomy if the bullae can be identified. Mechanical pleurodesis is usually performed by surgical pleurectomy or pleural abrasion. In this case report, we present a case of a young patient with spontaneous pneumothorax who needed a surgical intervention. We performed a new, innovative surgical technique for surgical pleurectomy where we used carbon dioxide for dissection of the parietal pleura (capnodissection). This technique may provide similar efficiency to the traditional procedure but with less risk of bleeding and complications.

## Introduction

Spontaneous pneumothorax (SP) is a condition in which pneumothorax occurs without trauma or iatrogenic cause. It can be classified as a primary SP if there is no obvious underlying lung disease. The most common cause is usually a small bulla or bleb in the lung [1,2]. Comparatively, secondary SP happens due to underlying lung diseases such as chronic obstructive pulmonary disease [3]. The new British Thoracic Society (BTS) guidelines advise surgical treatment for SP at initial presentation if recurrence prevention is deemed important (eg, patients presenting with tension pneumothorax or those in high-risk occupations). Elective surgery should be considered for patients with a second ipsilateral or first contralateral pneumothorax [4].

The surgical treatment that is recommended by the BTS guidelines for SP is surgical pleurodesis with or without bullectomy [4]. There are 2 common ways to perform surgical pleurodesis: the first one is surgical pleurectomy and the second one is pleural abrasion. Surgical pleurectomy is considered more efficient, but it can be associated with an increased risk of bleeding and infection [5]. The novel surgical technique that we provide here can give a similar success rate but with less risk of complications such as bleeding or infection.

## Case Presentation

Our patient was a young male individual who was previously healthy. He presented with a recurrence of an SP for the first time (2 SPs in total). The previous episode was treated conservatively 7 months prior, and his computed tomography scan for this episode showed that he had small apical bullae. The decision was made to list the patient for elective surgical treatment, and after discussion with the patient, he was listed for a pleurectomy and bullectomy.

**Figure 1:**
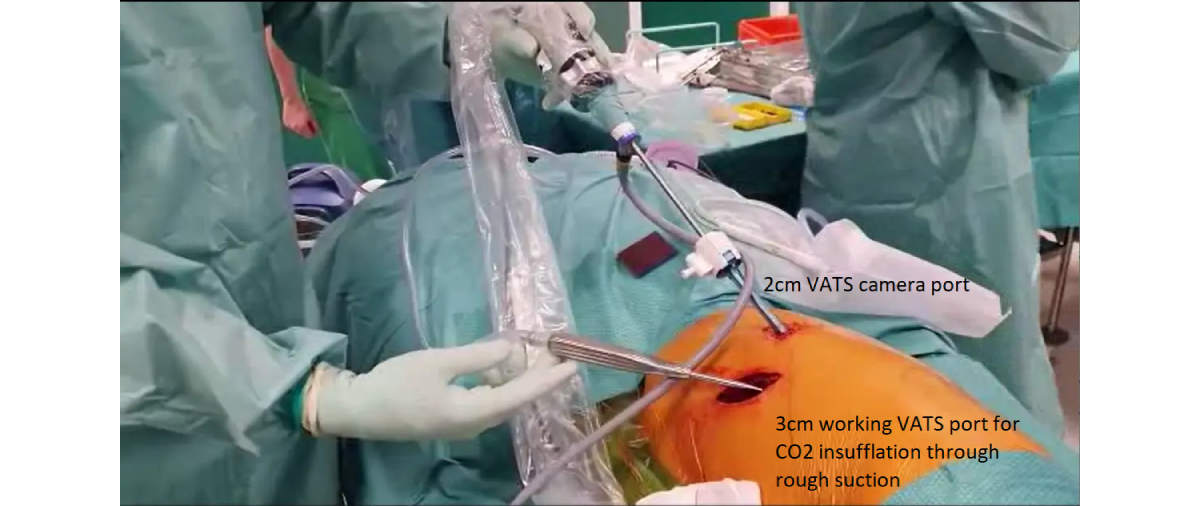
Video-assisted thorascopic surgery (VATS) ports setup and surgical instruments.

A standard anterior video-assisted thorascopic surgery (VATS) approach was taken. A small incision was made at the sixth intercostal space, and another small port site was created for the camera, which was later converted into the drain site (Figure 1). Carbon dioxide (CO_2_) insufflation at 6-8 mm Hg on high flow was used to achieve capnothorax. A small anterior VATS incision was made at the sixth intercostal space, and the dissection of the parietal pleura was performed extrapleurally using Roberts forceps with a traditional technique. The forceps were exchanged for a curved metal sucker, and the CO_2_ insufflation was attached at high flow and used to mobilize the whole parietal pleura, first from apex to inferior and then from posterior to anterior (Multimedia Appendix 1). The posterior parietal pleura was then excised off the ribs using thorascopic scissors 4 cm from the sympathetic chain posteriorly, 2 cm lateral to the internal mammary vein anteriorly, and 2 cm cranially to the diaphragm. Lastly, a bullectomy was performed using a manual stapler to excise the presumed culprit apical bullae seen on the computed tomography scan. Blood loss was minimal, approximately 50 mL, predominantly from VATS entry. Operative time was approximately 40 minutes. The postoperative care was routine, and the drain was removed after 48 hours. The patient was discharged on the third postoperative day.

## Ethical Considerations

On the day of the operation, the patient completed a written consent form. The patient kindly agreed to the recording of the procedure and the utilization of his nonidentifiable data for this case report and publication, which was further discussed between the patient and GQ. Consent was sought by GQ and given by those in the operating theater for recording of the technique and publication as a case report.

## Discussion

The use of CO_2_ in thoracic surgery has increased significantly with the growing use of a minimally invasive approach. Capnothorax leads to better visualization by collapsing the lung and reduces the rate of complications [6,7]. In our department, we usually use CO_2_ with robot-assisted thorascopic surgery and VATS for these reasons.

Surgical pleurodesis of SP is the recommended treatment in the BTS guidelines because it gives better long-term outcomes with less risk of recurrence in the future [4]. Surgical pleurectomy, in spite of it is efficiency, carries a risk of bleeding, infection, and reoperation [8]. Surgical pleural abrasion is another method that can be used for surgical pleurodesis. Chang et al [9] published the first systematic review and meta-analysis that compared surgical abrasion against surgical apical pleurectomy. They found that there is no difference in the recurrence, but pleural abrasion has a shorter length of stay in hospital, postoperative chest tube duration, and operative time and less surgical blood loss [9]. This may cause clinicians to consider a change of practice from surgical pleurectomy to abrasion. A systematic review of randomized controlled trials found that SPs managed with a chest drain alone had recurrence rates that ranged from 26.1% to 50.1%, whereas after VATS talc pleurodesis, these ranged from 0% to 3.2%. Alternative chemical pleurodesis can be achieved with tetracycline rather than talc, although recurrence rates were reported as ranging from 13% to 33.3% [10].

Our literature search did not find any studies in which capnodissection was used for pleurectomy as a treatment of SP. However, Dai, et al [11] recently published their findings for using CO_2_ for visceral pleurectomy and decortication in patients with malignant mesothelioma. They found that the positive pressure of CO_2_ can facilitate dissection of the visceral pleura, making the procedure easier while achieving an acceptable postoperative air leak and chest drain output. They concluded that capnodissection can be used in pleurectomy and decortication for patients with mesothelioma [11]. It should be taken into consideration that although the effect of capnodissection on gas exchange has not been rigorously studied, there is evidence to show that hypercarbia can result from CO_2_ insufflation for capnothorax during VATS or robot-assisted thorascopic surgery procedures [12]. This must be taken into account by surgeons and anesthetists when considering compensatory ventilator strategies, especially in patients with compromised gas exchange.

Our experience with the use of capnodissection for surgical pleurectomy was successful, and after 17 months from the procedure, the patient did not have any recurrence or complications. Moreover, this technique was not time-consuming (40-minute operative time), and the patient was discharged after 48 hours, with the surgeons noticing less pain in comparison to the traditional surgical pleurectomy, although pain is subjective. After VATS talc pleurodesis, the chest drain is typically removed no sooner than the second postoperative day, with discharge later that day. There is a theoretical risk of increased recurrence, as while the relatively atraumatic nature of this technique may reduce patient pain, it may also reduce the proinflammatory process required for pleurodesis and hence recurrence prevention [13]. More cases and longer follow-up are required to investigate the noninferiority of our technique to the traditional procedure.

## Conclusions

In this case, capnodissection of the parietal pleura was a novel, safe, and successful technique that may decrease the risk of bleeding and postoperative pain.

## Appendix

Multimedia Appendix 1 Video of capnodissection in action with surgeon narrative.

## Abbreviations

BTS: British Thoracic Society
CO_2_: carbon dioxide
SP: spontaneous pneumothorax
VATS: video-assisted thorascopic surgery

## Data Availability

Data regarding this paper have been deposited in the supplemental files. Corresponding author PR will enable further data sharing upon reasonable request.

## References

[1] Louw EH, Shaw JA, Koegelenberg CFN (2021). New insights into spontaneous pneumothorax: a review. Afr J Thorac Crit Care Med.

[2] Ghisalberti M, Guerrera F, de Vico A, Bertolaccini L, de Palma A, Fiorelli A, Paladini P, Ruffini E, Crisci R, Nosotti M, Mendogni P (2020). Age and clinical presentation for primary spontaneous pneumothorax. Heart Lung Circ.

[3] Nava GW, Walker SP (2022). Management of the secondary spontaneous pneumothorax: current guidance, controversies, and recent advances. J Clin Med.

[4] Roberts ME, Rahman NM, Maskell NA, Bibby AC, Blyth KG, Corcoran JP, Edey A, Evison M, de Fonseka D, Hallifax R, Harden S, Lawrie I, Lim E, McCracken D, Mercer R, Mishra EK, Nicholson AG, Noorzad F, Opstad KS, Parsonage M, Stanton AE, Walker S (2023). British Thoracic Society guideline for pleural disease. Thorax.

[5] Ocakcioglu I, Kupeli M (2019). Surgical treatment of spontaneous pneumothorax: pleural abrasion or pleurectomy?. Surg Laparosc Endosc Percutan Tech.

[6] Gallego-Poveda J, Guerra NC, Carvalheiro C, Ferreira H, Sena A, Junqueira N, Velho TR, Nobre Â (2017). Use of CO in video assisted thoracic surgery and single-lumen endotracheal tube-a new less invasive approach. J Thorac Dis.

[7] Brock H, Rieger R, Gabriel C, Pölz W, Moosbauer W, Necek S (2000). Haemodynamic changes during thoracoscopic surgery the effects of one-lung ventilation compared with carbon dioxide insufflation. Anaesthesia.

[8] Körner H, Andersen KS, Stangeland L, Ellingsen I, Engedal H (1996). Surgical treatment of spontaneous pneumothorax by wedge resection without pleurodesis or pleurectomy. Eur J Cardiothorac Surg.

[9] Chang J, Ratnaraj V, Fu V, Jiang M, Peri V, Nguyenhuy M, Antippa P (2023). Pleural abrasion versus apical pleurectomy for primary spontaneous pneumothorax: a systematic review and meta-analysis. J Cardiothorac Surg.

[10] Hallifax RJ, Yousuf A, Jones HE, Corcoran JP, Psallidas I, Rahman NM (2017). Effectiveness of chemical pleurodesis in spontaneous pneumothorax recurrence prevention: a systematic review. Thorax.

[11] Dai J, Liu M, Liu X, Li J, Jin K, Chen L, Bao M, Jiang G (2022). Carbon dioxide blower facilitates visceral pleurectomy in malignant pleural mesothelioma. Ann Thorac Surg.

[12] Tran DTT, Badner NH, Nicolaou G, Sischek W (2010). Arterial pCO2 changes during thoracoscopic surgery with CO2 insufflation and one lung ventilation. HSR Proc Intensive Care Cardiovasc Anesth.

[13] van den Heuvel MM, Smit HJ, Barbierato SB, Havenith CE, Beelen RH, Postmus PE (1998). Talc-induced inflammation in the pleural cavity. Eur Respir J.

